# Circadian rhythms and metabolism: from the brain to the gut and back again

**DOI:** 10.1111/nyas.13188

**Published:** 2016-09-02

**Authors:** Matthew R. Cribbet, Ryan W. Logan, Mathew D. Edwards, Erin Hanlon, Clara Bien Peek, Jeremy J. Stubblefield, Sridhar Vasudevan, Fiona Ritchey, Ellen Frank

**Affiliations:** ^1^ Western Psychiatric Institute and ClinicUniversity of Pittsburgh School of MedicinePittsburghPennsylvania; ^2^ Division of NeurobiologyMedical Research Council Laboratory of Molecular BiologyCambridgeUnited Kingdom; ^3^ Department of Medicine, Division of Endocrinology, Metabolism and Molecular MedicineNorthwestern University Feinberg School of MedicineChicagoIllinois; ^4^ Department of NeuroscienceUniversity of Texas Southwestern Medical CenterDallasTexas; ^5^ Department of PharmacologyUniversity of OxfordOxfordEngland

**Keywords:** circadian rhythms, metabolism, mood disorders, glucose, genetics

## Abstract

This paper focuses on the relationship between the circadian system and glucose metabolism. Research across the translational spectrum confirms the importance of the circadian system for glucose metabolism and offers promising clues as to when and why these systems go awry. In particular, basic research has started to clarify the molecular and genetic mechanisms through which the circadian system regulates metabolism. The study of human behavior, especially in the context of psychiatric disorders, such as bipolar disorder and major depression, forces us to see how inextricably linked mental health and metabolic health are. We also emphasize the remarkable opportunities for advancing circadian science through big data and advanced analytics. Advances in circadian research have translated into environmental and pharmacological interventions with tremendous therapeutic potential.

## Introduction

The International Scientific Group of Circadian Rhythm Experts (INSPIRE) was founded in 2013. The goal of this organization is to increase communication among international scientists studying circadian rhythms. Since 2013, annual meetings have provided a forum for basic and clinical scientists to share cutting‐edge translational research on the role of circadian factors in health and disease. Past meetings have focused on the role of the circadian system in brain function and development and how sleep–wake regulation relates to mood and anxiety. This report summarizes the scientific presentations of the third INSPIRE conference, which were centered around the theme of circadian rhythms and metabolism.

The third INSPIRE conference, held on April 16–18, 2015 in Viareggio, Italy and attended by 25 senior scientists from North America and Europe, focused on the relationships between circadian rhythms and metabolism and how these may relate to health and disease. The conference provided an opportunity for each senior scientist, who was accompanied by a junior investigator, to present data relevant to the theme of the meeting. Much of the richness of the conference came from the interaction between basic and clinical investigators to address the need for treatments for metabolic disorders and their comorbid diseases. Basic scientists are making clear progress in describing the bidirectional relationships between the circadian clock and metabolism. The challenge for clinical scientists is applying that progress to the development and testing of interventions. The conference was organized into six thematic sessions addressing (1) bipolar disorder as a model for the relationships among circadian dysfunction, metabolic dysfunction, and disease; (2) development, circadian rhythms, and metabolism; (3) the links between circadian function and glucose metabolism as seen through the lens of basic science; (4) circadian mechanisms in glucose metabolism in humans; (5) a debate on whether the clocks of peripheral organs are servants of the central clock of the suprachiasmatic nucleus (SCN); and, finally, (6) the relationship between metabolism and mood as seen in clinical studies. We summarize the presentations along with the debate, and conclude with a discussion of the basic and clinical perspectives on the future of translational research on circadian rhythms and metabolism and the implications for treatments.

## Bipolar disorder, circadian dysfunction, and metabolic dysfunction

### Overview

**Figure 1:**
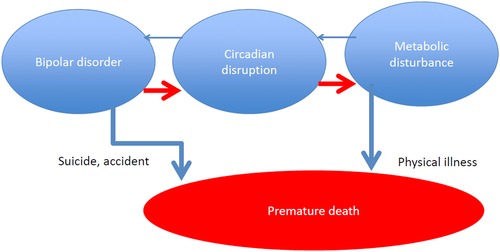
Circadian disruption could be a plausible link between the physical and mental aspects of bipolar disorder.

For the opening plenary of the third INSPIRE conference, Guy Goodwin of the University of Oxford discussed evidence that psychiatric illnesses are intimately involved with metabolic disorders, using bipolar disorder as a prototypic example (Fig. 1). He stressed that bipolar disorder is frequently accompanied by physical manifestations, such as obesity and cardiovascular disease, often leading to premature death. He suggested further that circadian disruption, either as an epiphenomenon or as a causal pathway, could be a plausible link between the physical and mental aspects of the disease and an emerging area for the development of novel therapeutic interventions.

### Bipolar disorder as a physical illness

Mortality data for patients with bipolar disorder indicate a reduced life span of 10–15 years, which is all the more remarkable when compared with the 6‐ to 10‐year reduction in life span attributed to smoking.^1^ This increased risk of mortality occurs across the life span,^2^ with 38% of the mortality arising from cardiovascular illnesses, a fourfold increased risk compared with the general population.^3^ In addition, metabolic syndrome is often comorbid with bipolar disorder, a twofold increased risk and >30% prevalence in untreated patients (45% in those treated with antipsychotics). Longitudinal studies have shown that patients with bipolar disorder are treated for diabetes and hyperlipidemia earlier in life than those in the general population, reflecting an inherent physical component of the illness.^4^ Interestingly, those who have diabetes show significantly worse outcomes for their mood symptoms.^5^ These findings suggest that bipolar disorder carries a pronounced metabolic burden that leads to physical illness beyond the associated mood symptoms. Studies on circadian rhythms, particularly those using mice with certain circadian gene mutations or environmental manipulations, have begun to demonstrate a primary pathological role of circadian disruption on metabolic, cardiovascular, and psychiatric diseases, including major depressive and bipolar disorders.^6^, ^7^ There appear to be complex bidirectional relationships between these diseases and circadian rhythms, such that circadian disruption can be a risk factor for particular diseases, while the chronicity of the disease can disrupt circadian rhythms, which may lead to an exacerbation of associated symptoms.

### Bipolar disorder and stress

Stress is a major precipitating factor for many psychiatric diseases, including bipolar disorder. In search of a link between metabolic disorder and comorbidity in those with bipolar disorder, Goodwin posed the question of whether mood dysregulation and the associated costs on daily lives could act as major stressors that contribute to the overall burden of the disease. Indeed, the literature on stressful life events and bipolar disorder highlights the many ways in which the illness is a provoker of stress through its negative impact on close relationships, employment, and housing. Furthermore, the extreme excursions of mood and accompanying excursions in hypothalamic–pituitary–adrenal axis parameters undoubtedly put stress on cellular systems. With the advent of smartphone‐based applications, an individual's mood, environment, and feeding and sleeping habits can be monitored more intensively and accurately. In such studies, stressful life events actually predicted both manic and depressive symptoms,^8^ and even more intriguing was the finding that individuals with bipolar disorder were more negatively affected by daily events, regardless of whether those events were positive or distressing.^9^ In dealing with these events, those with bipolar disorder required more effortful and cognitive self‐regulation of emotion,^9^ suggesting poor coping strategies and/or altered perception and stress reactivity. These are potential contributors to the exacerbation of mental and physical aspects of the disease, which undoubtedly have severe consequences on overall health.

Goodwin also proposed several biological mechanisms by which stress can affect the health of those with bipolar disorder. One of those mechanisms that is gaining research momentum is the role of oxidative stress in bipolar disorder. For instance, the enzymes responsible for reducing the accumulation of reactive oxygen species (ROS) in the cell are unchanged, while the consequences of increased ROS, particularly lipid peroxidation, are more severe in those with bipolar disorder.^10^ This prompted further investigation into the clearing mechanisms of ROS, specifically glutathione (GSH). The reduced form of ROS, which buffers ROS, was significantly reduced in bipolar patients.^11^ An elevated oxidative load in those with bipolar disorder links physical and psychiatric comorbidities. Although the precise mechanisms by which this may occur are unknown, the molecular clock could be primarily involved, especially since there is an extensive interplay between the clock and redox state.^12^, ^13^

### Genetics of bipolar disorder

Currently, we lack a clear understanding of the causes of bipolar disorder. Many, if not all, psychiatric disorders are heterogeneous and genetically pleiotropic, accompanied by poorly understood interactions with the environment. Heritability estimates of the disorder are as high as 75% in twin studies, suggesting a strong genetic component. However, due to disorder heterogeneity, gene pleiotropy, and small sample sizes, among other issues, genome‐wide association studies have failed to convincingly identify rare variants associated with bipolar disorder. Smaller studies with more targeted a priori approaches have identified genes involved in a number of cellular and molecular processes, such as calcium and glutamate receptor signaling, stress hyperreactivity, and the immune response. Each of these pathways has been linked to sleep, and they are slowly being implicated in setting the parameters of the molecular clock.^14^, ^15^ Moreover, there have now been over 50 human genetics studies linking polymorphisms in circadian genes with bipolar disorder, including *CLOCK* and others.^16^ The pleiotropic effects of these genes may explain the heterogeneity of the disorder in addition to the physical disease comorbidity.

### Conclusions

Goodwin concluded with a discussion of the necessity of early intervention focused on sleep and circadian rhythms as primary targets for behavioral and/or pharmacological treatments. Interventions capable of modulating or restoring alterations to sleep and/or circadian processes may mitigate the mood dysregulation and physical problems of bipolar disorder. In fact, chronotherapeutic interventions may have larger implications for general health and well‐being. The challenge is to clearly elucidate the role of circadian rhythms and sleep in bipolar disorder and how potential treatments may influence these systems to ameliorate symptoms and improve overall health.

## Development, circadian rhythms, and metabolism

### Overview

Michael Hastings (University of Cambridge) started the session by introducing the idea that circadian clocks are fundamentally designed to maintain good housekeeping in a cell and/or organism. Daily cycles of feeding and activity, along with molecular rhythms, maintain energy homeostasis and couple the circadian clock to metabolic systems. Interestingly, these relationships are bidirectional, in that changes in diet (e.g., high‐fat diets) can influence circadian patterns of feeding and activity and changes in cellular metabolic state and/or energetics can feed back to modulate the molecular clock. This session was focused on understanding the complex interplay between metabolic and circadian pathways at the molecular level.

### Circadian clock NAD^+^ cycle drives mitochondrial oxidative metabolism in mice

Clara Peek (Northwestern University) presented recent work from the laboratory of Joseph Bass uncovering a role for the molecular clock in the control of mitochondrial posttranslational modification and fuel selection.^17^ The molecular clock directly controls the rhythmic synthesis of the metabolic cofactor nicotinamide adenine dinucleotide (NAD^+^), which in turn modulates the activity of the histone and protein deacetylase sirtuin 1 (SIRT1). SIRT1 activity responds to and controls cellular metabolism and provides feedback to the molecular clock.^18^, ^19^, ^20^, ^21^ Thus, the molecular clock regulates metabolic function, in part, through NAD^+^ and other factors, to modulate SIRT1 activity, which can feed back to influence the clock. Peek investigated whether clock‐driven NAD^+^ cycles in peripheral tissues could affect the activity of mitochondrial NAD^+^‐dependent sirtuin enzymes that are important for fuel utilization and energy balance.

The SCN controls feeding and activity rhythms producing 24‐h variation in the nutrient state of peripheral tissues. Local clocks in peripheral tissues also directly control metabolic processes, including nuclear hormone receptors and NAD^+^‐dependent sirtuin enzymes. One challenge in the field has been to uncouple the role of the central clock and feeding behavior from tissue‐specific molecular clocks for nutrient utilization and energy homeostasis. To circumvent the effects of feeding behavior, Peek and colleagues used separate cohorts of mice, fasted for equal amounts of time, and collected liver tissue to measure fatty acid utilization.^17^ Fatty acid oxidation displayed a pronounced rhythm paralleling NAD^+^ synthesis in the liver, suggesting that clock‐controlled NAD^+^ synthesis affects mitochondrial function. In support of this, Peek demonstrated that respiration of intact liver mitochondria was disrupted in *Bmal1* mutant mice. Mitochondrial function is therefore controlled by the molecular clock through a mechanism independent of mitochondrial biogenesis or turnover. Enzymes important for fatty acid utilization and oxidative metabolism were also hyperacetylated in *Bmal1* mutant liver. These enzymes are target proteins of the NAD^+^‐dependent deacetylase SIRT3, which localizes to mitochondria, suggesting a clock‐induced defect in mitochondrial SIRT3 deacetylase activity. Injection of a precursor to NAD^+^ essentially bypasses the clock‐controlled step in the synthesis of NAD^+^ and rescues the mitochondrial defects observed in circadian mutant mouse liver. Together, these findings describe a mechanism for the circadian regulation of fuel utilization via liver‐generated cyclic NAD^+^ synthesis and mitochondrial posttranslational protein modification.

Peek ended the presentation with more recent studies demonstrating similar effects of clock disruption on NAD^+^ and oxidative metabolism in mouse skeletal muscle, raising the possibility that similar circadian mechanisms of mitochondrial regulation exist in other metabolic tissues.^17^ In addition, mitochondrial signaling factors may feed back to modulate clock‐controlled gene transcription. Indeed, a number of mitochondria‐derived nutrients, including ROS (organic acids,^22^ fatty acids,^23^ and NAD^+^/NADH^24^), have been shown to affect nuclear function. More precise metabolomics and genomics techniques will be necessary to understand the extent of circadian control of fuel selection and utilization and the reciprocal relationships between peripheral clocks and nutrient‐responsive transcriptional networks. It will be important to explore whether disrupted clock‐controlled mechanisms are a primary mechanism of tissue pathology associated with metabolic disorders and other diseases.

### Redox and metabolic oscillations in the clockwork

Akhilesh Reddy (University of Cambridge) began his presentation with an overview of the concept of resonance to understand the connections between metabolism and circadian clocks. Resonance refers to the idea that all organisms are designed to align their inside world (i.e., internal physiological processes) with the demands they face from the outside world. For example, our relatively recent human ancestors (50,000 years ago) needed to eat and forage for food during the day and fast and sleep at night. However, modern schedules often disrupt our outside worlds by exposing us to light and feeding at nonoptimal times, while our inside worlds remain tied to the phase of the internal clock. On the basis of this hypothesis, it is possible that the dissonance between internal physiology and external behavior caused by our modern lifestyles contribute to metabolic dysfunction and disease.

Virtually every cell in the body expresses the canonical circadian genes of the molecular clock, except for red blood cells (RBCs). Reddy presented recent findings from his laboratory showing the presence of nontranscriptional circadian rhythms in anucleate human RBCs driven by the oxidation status of the peroxiredoxin antioxidant proteins.^26^ Hyperoxidation of both peroxiredoxin and reduction factors, including NADH and NADPH, displays rhythmicity in human RBCs cultured *ex vivo*. Oxidative and reductive (redox) balance rhythms are independent of the core circadian transcription–translation feedback loop (TTFL) and have been shown more recently to be highly conserved throughout all kingdoms of life.^12^

**Figure 2:**
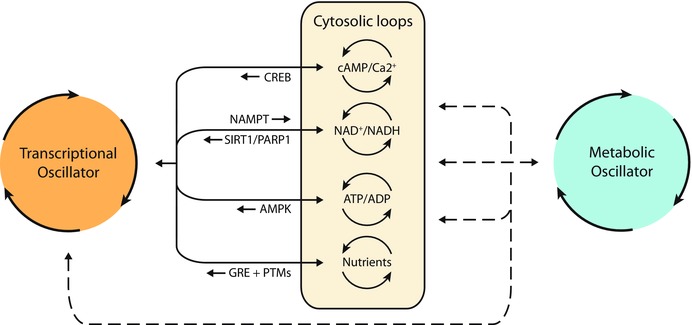
Transcriptional, cytosolic, and metabolic cycles in human red blood cells. Transcription–translation feedback loops and redox cycles coexist and have extensive cross talk through redox cofactors, such as NADH and NADPH. NADH and NADPH can alter the phase and amplitude of circadian gene bioluminescent reporters in isolated red blood cells. Reprinted with permission from Ref. 25.

In other cells, the TTFL and redox cycles coexist and have extensive cross talk. Reddy proposed that these two oscillators are linked through redox cofactors, such as NADH and NADPH, which can alter the phase and amplitude of circadian gene bioluminescent reporters in isolated cells.^26^ The findings support the idea that NADP(H) redox cycles may interact with the core TTFL oscillator (Fig. 2).

Using both *in vitro* and *in vivo* models, Reddy then presented pharmacological and genetic studies revealing novel mechanisms by which the pentose phosphate pathway can regulate the TTFL in single neurons of the mammalian SCN and behavioral activity rhythms of *Drosophila melanogaster*.^26^ These studies provide novel perspectives on the coupling of metabolic and circadian pathways via direct regulation of the TTFL by redox cofactors. The pentose phosphate pathway is also capable of metabolizing glucose. Interestingly, other factors involved in glucose metabolism influence the TTFL, while the circadian system can directly or indirectly influence glucose metabolism.

## The links between circadian function and glucose metabolism

### Overview

Christian Boitard (INSERM, Paris) introduced the session by reiterating that rhythmic biological processes are the result of internal and external processes. Investigating how these inner and outer worlds interact to coordinate rhythms across the entire organism (i.e., multiple organ systems and cell types) requires a systems biology approach. Considering how these systems interact in the rhythmic milieu of the organism will improve medical treatment and should aid in the development of novel, more efficacious therapeutics and interventions.

### Nuclear receptors and the control of lipid and glucose metabolism and cardiovascular function in humans

Bart Staels (University of Lille) discussed the role of bile acids in metabolism. The enterohepatic cycling of bile acids includes production in the liver as derivatives of cholesterol, storage in the bile, secretion into the intestines, and uptake, after which the majority that is not secreted recycles back into the liver. Bile acid synthesis is regulated by circadian rhythms. For instance, there are diurnal variations in bile acid concentrations, and mutation of the *Clock* gene interrupts bile acid homeostasis in mice.^27^, ^28^ In human serum, the bile acid precursor C4 is rhythmic, with the highest peaks during midday and following an evening meal, which is antiphasic to cholesterol synthesis.^29^ Moreover, the expression of cholesterol 7‐α‐hydroxylase (CYP7A1), an enzyme involved in the synthesis of bile acid, is rhythmically regulated by transcription factors of the molecular clock. Thus, bile acid synthesis and metabolism are modulated by both systemic and local circadian mechanisms, in addition to behavioral and environmental factors (e.g., feeding schedules).

Bile acids, including the TGR5 receptor and farnesoid X receptor (FXR) pathways, act as signaling molecules to modulate glucose, lipid, and energy homeostasis. Recent studies have investigated the role of bile acids in the transition from insulin resistance to type 2 diabetes. Bile acids are increased in those with insulin resistance or type 2 diabetes.^30^, ^31^ Bile acid sequestrants are used to treat diabetes by binding bile acids in the intestine and interrupting the diurnal enterohepatic circulation. This observed improvement of metabolic control may involve cross talk between the liver, intestine, and pancreas and bile acid signaling. The bile acid receptor, also known as FXR, is a nuclear receptor that has a role in glucose utilization in the postprandial phase, and *Fxr* knockout mice have delayed intestinal glucose absorption.^32^ In addition, bile acids increase production and secretion of the incretin glucagon‐like peptide 1 (GLP‐1), which increases insulin release from pancreatic β cells. TGR5 receptor activation induces intestinal GLP‐1 secretion, and FXR receptors on human L cells may also be involved. FXR may influence glucose utilization by the L cell, which is important in triggering the secretion of GLP‐1, although the experiments are still ongoing. Staels also addressed the connection between bile acid metabolism and gut microbiota. Secondary bile acids are produced directly by gut microbiota. The gut microbiota influences the production of ligands for FXR and TGR5.^33^ Together, these findings demonstrate the role of bile acid signaling in glucose metabolism and sensing across the liver, intestine, and pancreas.

### Circadian posttranscriptional control of metabolism

Carla Green (University of Texas Southwestern Medical Center) highlighted the pervasiveness and functional importance of circadian rhythms at the molecular level by discussing how thousands of genes and proteins are expressed rhythmically across many tissues. Many of these genes are driven by CLOCK/BMAL1 transcriptional regulation, which can lead to protein rhythms, many of which are intimately involved in glucose metabolism.^34^ However, recent findings from her laboratory have shown that rhythmic mRNA expression is also regulated by posttranscriptional processes involving deadenylation in the liver,^35^, ^36^ specifically within mitochondria.^37^ Interest has grown regarding rhythmic polyadenylation processes in mitochondria as a result of recent reports of circadian oscillations of oxidative phosphorylation, β‐oxidation, and NAD^+^ cycling.^38^

Green discovered that the primary mechanism by which circadian rhythms affect polyadenylation is through the rhythmic expression of the deadenylase Nocturnin. Unlike other canonical deadenylase enzymes, which are either constitutively expressed or display low‐amplitude rhythms, Nocturnin displays robust, high amplitude expression rhythms in the liver. Nocturnin null mice (*Noc^−/−^*) are resistant to diet‐induced obesity and hepatic steatosis, suggesting a key role for Nocturnin in liver metabolism.^39^ *Noc^–/–^* mice have disrupted lipid processing in the gut and liver, likely due to altered polyadenylation of several key processing enzymes.^40^ Thus, posttranscriptional control of mRNA, including for enzymes involved in liver metabolism, is regulated by the molecular clock. In fact, recent findings indicate that the mammalian molecular clock drives mRNA rhythms across multiple levels via *de novo* transcriptional, posttranscriptional, and epigenetic mechanisms.^35^ A deeper understanding of these mechanisms, such as those downstream of Nocturnin, may aid in the development of treatments for metabolic disorders associated with disrupted rhythms. Several groups have started to investigate similar metabolic phenomena in humans.

### Circadian rhythms of glucose metabolism in humans

Eve Van Cauter (University of Chicago) reviewed the relationships among sleep, circadian function, and glucose metabolism and began with an explanation of a phenomenon identified in the 1960s, referred to as “afternoon diabetes.” Investigators noticed that, in healthy young adults, peak glucose levels were higher when an oral glucose tolerance test was performed in the afternoon (4 p.m. or later) than if the same individuals were tested in the morning. This was one of the first indications that glucose tolerance was not constant across the 24‐h cycle. Subsequent studies revealed that, under conditions of constant glucose infusion, blood glucose levels display a profile that is modulated by circadian rhythmicity and sleep.^41^ The impact of sleep, sleep disturbance, and circadian dysfunction on diabetes risk gained scientific and public interest. Data collected by the Centers for Disease Control (CDC) in the United States have shown that regions with a higher percentage of obesity and diabetes overlap with those that report elevated levels of insufficient sleep. In the laboratory, Spiegel *et al*.^42^ assessed diabetes risk in healthy lean volunteers via an intravenous glucose tolerance test following either short sleep (five nights of 4 h/night) or the rested condition (five nights of 12 h/night).^42^ Both insulin sensitivity and disposition index, a marker of diabetes risk, were significantly decreased following short sleep in healthy volunteers. These findings have been replicated in subsequent studies with varying degrees of sleep time and quality.^43^, ^44^, ^45^, ^46^, ^47^, ^48^

A recent study reported that sleep loss may directly affect peripheral tissues, specifically adipocytes.^46^ Subcutaneous abdominal fat was collected from healthy lean volunteers following either four nights of short sleep (4.5 h/night) or normal sleep (8 h/night) in a randomized crossover design. The adipocytes were challenged with increasing concentrations of insulin, and levels of phosphorylated Akt (pAkt), a critical step in the insulin‐signaling pathway, were measured by immunoblotting. Following short sleep, adipocytes exhibited a 30% reduction in insulin sensitivity compared with those collected following normal sleep; following short sleep, higher concentrations of insulin were necessary to elicit the same pAkt response.^46^ These data revealed that sleep is not only beneficial for the brain but also for peripheral function (i.e., adipocytes).

Having reviewed the detrimental effects that sleep loss can have on metabolism, Van Cauter then addressed whether sleep extension and/or improvement in sleep quality can have beneficial effects on glucose metabolism. A correlate of the obesity epidemic is the increased prevalence of obstructive sleep apnea (OSA). OSA is associated with intermittent hypoxia and results in reduced sleep time, sleep fragmentation, and decreased sleep intensity. Punjabi and Beamer^49^ demonstrated that, in nondiabetics, the severity of OSA is associated with a decrease in insulin sensitivity. In individuals with diabetes, multiple studies have reported a high prevalence of OSA; approximately two out of three diabetic patients will also have OSA.^50^, ^51^, ^52^, ^53^, ^54^ An interesting question is whether treatment of OSA can affect the severity of diabetes and impaired glucose metabolism. Encouragingly, a recent laboratory study revealed that 8 h/night for 2 weeks of continuous positive airway pressure (CPAP) treatment for OSA improved glucose metabolism in prediabetics as compared with placebo.^55^ The studies examining the impact of treating OSA on glucose metabolism in diabetics are still ongoing.

With respect to circadian misalignment, Van Cauter discussed the multitude of studies that have associated shift work, both cross‐sectionally and prospectively, with adverse health outcomes. Most specifically, one study of 1351 European men detailed an increased risk of metabolic syndrome, weight gain, and diabetes associated with shift work.^55^ Since shift workers invariably also experience sleep loss, a laboratory study attempted to tease apart the impact of sleep loss and/or circadian misalignment on glucose metabolism and revealed that, when sleep restriction and circadian misalignment were combined, the decrease in insulin sensitivity was nearly double that observed with nocturnal sleep restriction alone.^47^ Moreover, chronotype is associated with glycemic control in type 2 diabetics; individuals with a later chronotype (associated with a higher degree of circadian misalignment) had higher HbA_1C_ levels.^56^ These data reveal that circadian misalignment can adversely affect glucose metabolism, and, furthermore, when combined with sleep restriction, a common occurrence in circadian misalignment, this affect can be exaggerated. Finally, Van Cauter demonstrated that sleep and circadian issues can be significant risk factors in regard to diabetes and, in fact, may be stronger risk factors than those typically associated with obesity and diabetes, including sedentary lifestyle.

### Separate effects of circadian system and circadian misalignment on glucose metabolism in humans

Frank Scheer (Harvard University) has investigated the impact of simulated circadian misalignment and desynchrony on metabolism in humans. The hierarchy of the circadian system is critical for circadian modulation of peripheral oscillators by the central pacemaker of the SCN. However, multiple *zeitgeibers* can differentially influence rhythms across these tissues (e.g., photic input to the SCN versus nutrient signals to the liver and other peripheral tissues). Entrainment signals that are out of phase or unsynchronized with one another, such as occurs in the case of shift work, may promote certain disease states. Investigating the relative contributions of the circadian system and the environmental or behavioral factors to circadian alignment could enhance our understanding of the disease state.

To study the effects of circadian misalignment on disease‐related processes, Scheer *et al*.^57^ employed a forced desynchrony protocol, which distributes the various behavioral cycle components equally throughout the circadian day. Using 3 days of a 28‐h forced desynchrony paradigm in otherwise healthy human subjects, postprandial glucose levels were altered following a morning or evening meal. Postprandial insulin secretion was higher without changes in insulin secretion, suggesting an alteration in insulin sensitivity. After cessation of the study, subjects returned to normoglycemia. The relative contribution of sleep disruption to these results was not directly assessed, but recent work demonstrated that the consequences of circadian misalignment on insulin sensitivity may be separate from sleep–wake disruptions.^47^

Other studies have used approaches that more closely mimic the 24‐h misalignment that people encounter. To accomplish this, Scheer and his group designed a forced desynchrony crossover protocol in which the metabolic parameters were measured when subjects’ behavioral cycles were either aligned to their circadian cycles or misaligned through a rapid, 12‐h shift of the behavioral cycle.^58^ This allowed them to probe the relative effects of circadian phase and circadian misalignment on metabolism, independent of behavioral feeding/fasting and sleep/wake cycle.

It has been shown that the degree of glucose tolerance varies with the time of day. Glucose tolerance appears to respond more efficiently to a morning meal than an evening meal, suggesting that the temporal variation may be controlled by circadian phase. Moreover, circadian misalignment has minimal consequences on glucose tolerance. Thus, there seems to be robust circadian control of insulin concentration, which is reflective of β cell function and insulin sensitivity. Indeed, the acute postprandial insulin response is much greater in the morning than in the evening of the circadian day.

Melatonin is released rhythmically in many mammals to synchronize oscillators in the brain and peripheral tissues. Alterations in rhythmic melatonin release are hypothesized to have profound consequences on disease states, such as cancer and psychiatric disorders. Melatonin also seems to play a major role in glucose tolerance of peripheral tissues. Exogenous melatonin given to patients during the day (when levels are usually low) impairs glucose tolerance.^59^ When circadian and behavioral cycles are misaligned, Sheer and colleagues discovered that melatonin rhythms remain present yet significantly dampened.^58^ These initial findings imply that if meals are consumed at circadian phases when melatonin is high, such as the evening, then glucose processing may be impaired, and mistimed meals may lead, over extended periods of time, to metabolic disorders, obesity, and other diseases. Interestingly, polymorphisms of the melatonin 1b receptor gene predicted the risk of diabetes in humans.^60^

Circadian alignment of biological (internal) and environmental (external) cycles is important for proper glucose metabolism. Future studies could examine the importance of different types of nutrients consumed at different times of day and under different behavioral cycles. In healthy subjects, tolerance for carbohydrates is more efficient during the biological morning than evening, but the temporal processing of lipids and proteins needs further examination. It will also be important to explore how patients in disease states like diabetes handle meals/nutrients at certain times of day under circadian alignment and misalignment conditions.

### An atlas of circadian gene expression: implications for biology and medicine

There has been an explosion in high‐throughput, massive data–generating technologies in biomedical research. These techniques are often employed as a means of unbiased discovery to obtain information on a wide array of biological processes across many tissues under multiple experimental conditions. The data are further complicated by repeated sampling across circadian cycles. If the data are to be used for translational implementations, such as interventions or therapeutic development, interpretations need to be guided by clear, concise, and accessible analyses and databases. John Hogenesch (University of Pennsylvania) discussed a few of the difficulties when implementing, analyzing, and disseminating large data sets and how newly developed tools should help meet some of these challenges.

There is tremendous interest in determining whether the portion of the transcriptome is rhythmic across every tissue in the body and whether these pathways are involved in disease processes. Rhythmically expressed genes identified to be common across many tissues are more likely to be involved in core molecular clock regulation, whereas those specific to a given tissue could be considered to be clock‐controlled genes or outputs of the clock. In an effort to more accurately and robustly identify rhythms of the transcriptome, Hogenesch and colleagues curated a database of gene expression profiles, integrating findings from his own laboratory and others using microarray and RNA sequencing data from multiple mouse tissues and cell lines (CircaDB: http://bioinf.itmat.upenn.edu/circa/). The results suggest that circadian regulation of the transcriptome is complex, with at least 43% of the mouse transcriptome cycling in at least one tissue, with a majority of these genes cycling in a tissue‐ or cell type–specific manner. These oscillatory patterns show different phasing across tissues. For example, the acrophase of gene expression in the liver is during the late‐night, anticipating‐sleep/rest phase of the mouse, while genes in the lung cycle around the light–dark transition, likely anticipating the onset of activity or wakefulness.

Being able to interpret and apply the data to questions regarding biological significance and relatedness to disease is more difficult. For example, what is the significance of a cluster of genes oscillating in phase in the liver but at different phases in the lung? One approach is to bin data within different phasing clusters (i.e., genes clustered together over a 4‐h period at different circadian times). Hogenesch suggested that a unbiased approach to binning this temporal data is to use gene set enrichment analysis (GSEA).^61^ The procedure detects groups of genes that change together over a range of phases, though it is limited in that it cannot effectively handle the periodic nature of circadian data (i.e., time is not necessarily linear, as time 0 is the same as time 24‐h in a circadian day). To extend the utility of the GSEA approach for circadian analyses, Hogenesch included the Kuiper tests among other analyses, with statistically uniform power across the cycle (referred to as phase set enrichment analysis (PSEA)).

**Figure 3:**
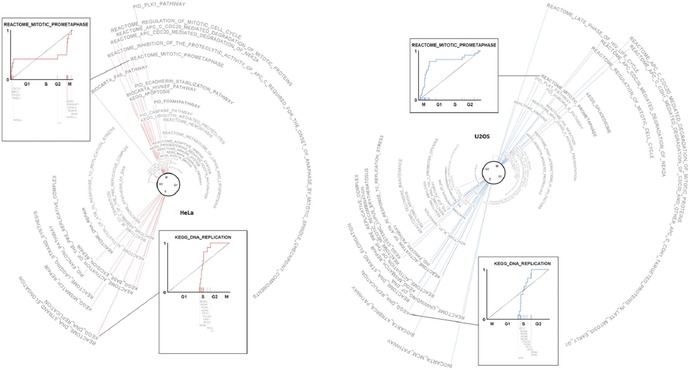
An atlas of circadian gene expression across the cell cycle using phase set enrichment analysis.

Using PSEA or other strategies to identify and understand how genes and clusters of genes are regulated by circadian phase, we can begin to design translational studies employing chronotherapy for the treatment of different diseases and conditions (Fig. 3). For example, the circadian timing of drug delivery during cancer treatment has been appreciated for some time.^62^ Other fields are poised to benefit from these types of databases as well, as circadian rhythms are thought to be putative contributors to obesity, diabetes, depression, anxiety, addiction, and many other diseases. Interestingly, many of the best‐selling pharmaceuticals target genes and mechanisms of the circadian system. It is possible that the knowledge of when particular targets are expressed in which tissue may aid in the development of more effective treatments and drug delivery. Standardized analyses, such as PSEA, and curated, accessible databases will better inform researchers and clinicians on the specific and global effects of different drug compounds and therapies.

## The INSPIRE 2015 debate, resolved: glucose metabolism needs the brain; the clocks of peripheral organs are servants of the suprachiasmatic nucleus

For the third consecutive INSPIRE conference, a debate occurred over a timely and most relevant topic in circadian biology, led by leaders in the field. The debate was chaired by David Kupfer (University of Pittsburgh), while Gene Block (University of California, Los Angeles) and Andrew Loudon (University of Manchester) presented the affirmative and negative positions on the topic, respectively.

### Affirmative: peripheral clocks are servants of the suprachiasmatic nucleus

Block's main argument hinged on clarifying the role of the SCN in regulating peripheral oscillators. He presented several pieces of historical evidence accompanied by more recent studies strongly suggesting that peripheral oscillators, especially those controlling metabolic pathways, are servants, not slaves, of the SCN. Peripheral oscillators are coupled to the central circadian pacemaker of the SCN by direct and indirect pathways. It is through these pathways that the SCN may set and maintain the phase, period, and amplitude of these servant oscillators. Block posited that this system is analogous to a set of pendulums, where the heavier the pendulum, the SCN in this case, the more important and influential, while the length of the arm represents the period, and these arms are coupled by rubber bands, allowing these other pendulums to move within the systems limits. He urged the audience to broaden their conceptualization of the coupling between these central and peripheral oscillators—even servants are loosely tied to their jobs.

#### SCN as pacemaker for the circadian system: multiple control mechanisms of peripheral glucose metabolism

Synchrony between the environment and the circadian system regulates the daily rhythms of many biological processes, such as sleep–wake cycles and feeding. Light is the main environmental input to entrain the master circadian pacemaker of the SCN to 24‐h rhythms. The SCN then sends outputs to other areas of the brain and peripheral tissues via indirect (e.g., sleep–wake, arousal, or temperature rhythms) and/or direct (e.g., neural or humoral signals) pathways to provide coherence across multiple physiological systems.

SCN outputs control the phase and amplitude of rhythms in many peripheral tissues via several systems. Using *mPer2^luciferase^* (PER2::LUC) transgenic mice, Yoo *et al*.^63^ demonstrated phase coherence and reproducible phase relationships between peripheral tissues under entrained light–dark (LD) conditions *in vitro*. Under dark–dark conditions, PER2 rhythms were dispersed slightly between animals depending on their individual free‐running periods, and phase coherence was retained. SCN lesions promoted phase desychrony among peripheral tissues, including the kidney, lung, and liver, which became even more apparent after a long period (32 days) in constant darkness, suggesting that the system has lost coherence.^63^ Studies examining PER2::LUC rhythms *in vivo* suggest an even greater involvement of the SCN to control peripheral rhythms. SCN lesions disrupted *in vivo* phase coherence of peripheral rhythms, similar to the *in vitro* findings, and, furthermore, rhythms were significantly dampened in peripheral tissues, including the liver.^64^ Even more intriguing is that, when these aperiodic tissues due to an SCN lesion are removed from the animal, the transfer to an *in vitro* preparation restores rhythmicity,^64^ providing some explanation for the inconsistent findings between *in vitro* and *in vivo* studies. Therefore, many peripheral tissues may need the SCN to maintain phasing and also some level of daily input to sustain rhythm amplitude and robustness.

Anatomical tracing studies revealed direct connections to the liver from the SCN via the autonomic nervous system (ANS).^65^, ^66^ The SCN sends both stimulatory (glutamatergic) and inhibitory (GABAergic) inputs to the same preautonomic sympathetic neurons in the paraventricular nucleus of the hypothalamus. Continuous glutamatergic input to these neurons is directly regulated by rhythmic GABAergic input, which in turn controls the phasing and timing of ANS communication to the liver and rhythmic hepatic glucose production.^67^ Electrical stimulation of the SCN induces hyperglycemia, which can be prevented by blocking adrenergic ANS input to the liver.^68^, ^69^ A parallel pathway from the SCN to orexin‐producing neurons of the perifornical area is another potential pathway for circadian regulation of hepatic glucose production.^70^, ^71^, ^72^

Early studies strongly implicated the SCN in regulating peripheral glucose metabolic rhythms. Lesions of the SCN completely abolished the rhythms of plasma insulin and glucose.^73^, ^74^ More recent studies have used daily scheduled feeding regimens to more clearly define the role of the SCN in controlling glucose rhythms. Interestingly, scheduled feeding during the night phase of the LD cycle in rats with SCN lesions induced higher plasma insulin and glucose levels compared with the day phase, which recapitulated the daily rhythms observed in SCN‐intact animals.^75^ SCN lesions also lead to severe hepatic insulin resistance.^76^ Further support of circadian regulation of glucose metabolism comes from fasted animals, where glucose rhythms are entirely maintained.^75^

The SCN may inhibit phase shifts to nonphotic stimuli, such as food availability, under normal conditions. Mice placed on a food availability schedule 180° out of phase with the LD cycle (i.e., food is available during the day) gradually shifted BMAL1::LUC hepatocyte rhythms over an 8‐day period, whereas hepatocyte rhythms shifted in almost half of that time in SCN‐lesioned mice.^77^ When feeding rhythms are out of phase with the rest of the animal, the SCN uses signaling pathways to attenuate entrainment of hepatic oscillators to feeding schedules, providing further support for direct SCN regulation of peripheral metabolism.

These results and other studies suggest that there are some unresolved issues, particularly how peripheral oscillators provide feedback to modulate SCN function and the rest of the circadian system. Block presented several examples related to this point. A major assumption throughout many of these experiments is reflected by the use of rest–activity cycles as the main (or only) marker of endogenous SCN phase and period. The early findings by Aschoff^78^ demonstrate that rhythms can be dissociated, some under constant conditions, from one another (i.e., rest–activity cycles may begin in phase with core body temperature rhythms but eventually desynchronize and run at a shorter period). In mice, molecular rhythms in the SCN and behavioral rhythms can be dissociated under shorter (t‐20) and longer (t‐28) light schedules. As expected, long and short photoperiods resulted in longer and shorter free‐running behavioral rhythms, respectively.^79^ However, *in vitro* PER1::LUC rhythms in the SCN were negatively correlated with behavioral rhythms, such that longer free‐running rhythms following long t‐cycles resulted in shorter PER1 rhythms, and the opposite was observed for shorter t‐cycles.^79^ It is intriguing to hypothesize that the mismatch between molecular and behavioral rhythms is due to plasticity within the circadian system, where SCN and extra‐SCN oscillators are regulating different phases and/or periods of molecular, physiological, and behavioral rhythms.

It is possible that extra‐SCN oscillators are feeding back to the SCN to modulate or dissociate coherence across the circadian system. There is strong evidence that peripheral oscillators provide some level of feedback to the SCN to impede the overall flexibility and to promote coherence across the circadian timing system. Meijer and colleagues phase shifted rats by a 6‐h advance in the light schedule to investigate *in vitro Per1* rhythms and *in vivo* and *in vitro* electrical rhythms in the SCN at 1, 3, or 6 days following the shift.^80^ Intriguingly, both *Per1* and electrical rhythms from *in vitro* SCN recordings reflected large phase shifts (∼3–6 h) even 3–6 days following the initial shift, whereas *in vivo* electrical recordings and behavior showed no such shift.^80^ Other factors, such as those resembling extra‐SCN oscillators or other mechanisms, may be actively feeding back to the SCN, even at the level of the output (e.g., locomotor activity rhythms), to attenuate the response or to pull the SCN back into its old phase. Despite the ability of scheduled feeding to entrain hepatic rhythms independent of the SCN and light–dark cycle, these rhythms immediately return to the SCN‐driven phase when the animal is returned to constant darkness.^81^ Hence, food entrainment may uncouple hepatic and SCN rhythms, but, under normal circumstances, this is likely driven by the SCN through control of feeding circuits.

Despite his strong stance, Block urged that we should remain cautious of our interpretation of the data thus far and focus on designing future studies to not only tackle the inconsistencies in the literature, but also to provide new insights into the role of the SCN and circadian system in peripheral rhythms and physiology.

### Opposed: the hegemony of the suprachiasmatic nucleus

Loudon argued that the field as a whole has a limited view of how the circadian system regulates metabolic systems, primarily owing to our mammalian‐centric investigations. More subtly, his argument addressed the inherent need for the scientific community to remain interested in the evolutionary and adaptive qualities of the circadian system to environmental stimuli and the response requirements of an organism to maintain optimal homeostasis and survival. Actively employing this conceptual framework may lead to more creative and novel discoveries to identify the mechanisms by which the circadian system, either with or without SCN involvement, controls metabolic systems and feeding behavior.

#### Lessons from nature: entrainable and adaptive oscillators of diverse species

A majority of the basic mechanism studies are conducted using standard laboratory‐bred mice. Mice have developed robust circadian oscillators that entrain their behavior to changing LD cycles. The mouse relies heavily on daily light input to the SCN to regulate rhythms of many prosurvival behaviors, such as feeding. In other organisms, including some fish species, photic input completely bypasses the central nervous system to entrain the body. For example, both zebrafish and Somalian cavefish are transparent, light‐permeable organisms, yet these species entrain quite differently to environmental light input. Almost every cell of the zebrafish is a light sensor and has the potential to entrain to photic input.^82^, ^83^, ^84^ Somalian cavefish, on the other hand, do not entrain to light, but will readily entrain to feeding schedules,^82^ suggesting that the circadian system has adapted to a perpetually dark ocean environment by entraining to food availability rather than light. Zebrafish can also entrain to feeding schedules.^82^ Thus, feeding rhythms entrain peripheral metabolic rhythms independent of the SCN.

The Svalbard reindeer is another example of adaptation to a nonrhythmic environment. The Svalbard reindeer live ∼800 miles from the North Pole, where they are exposed to continuous light during the summer and continuous dark during the winter. These animals have a robust metabolic cycle, profound glucose sensing, and modify their voluntary food intake depending on the time of year, accompanied by massive fat deposition—more than a fivefold increase in food intake during the summer months.^85^ Over the course of the year, circadian organization of their behavior is virtually absent, even during the middle of the winter, yet they display very strong rumination ultradian rhythms in order to feed.

The melatonin system seems to be the synchronizing signal of seasonality. In humans, the SCN drives the melatonin rhythm from the pineal gland, with light having a dual effect: it both synchronizes melatonin rhythms and inhibits melatonin production. In Svalbard reindeer, there is no capacity for the system to produce a melatonin rhythm under the constant darkness conditions of the winter season.^86^, ^87^ Laboratory studies have shown that melatonin levels respond to the onset of darkness, which gradually dissipates over several days into constant darkness.^86^ Melatonin levels are also driven under short t‐cycles (5 h), only elevating during the transition to darkness and quickly subsiding after the transition to light, suggesting that the SCN‐driven circadian system is not involved. Loudon posits that there is little selective advantage to maintain strong internal clocks in nonrhythmic environments, especially when survival depends on high seasonal awareness and preparation.

Loudon went on to present a few experiments to address the matter that “there is little to no evidence yet that the peripheral clock is important in *normal* physiology.” He suggested that one strategy would be to utilize animals with dramatically altered free‐running molecular and behavioral rhythms, such as the casein kinase 1 epsilon (CK1ε) *tau* mutant mouse. The *tau* mutation of the Syrian hamster was the first circadian mutation discovered.^88^ Loudon and colleagues developed a mouse with the Hamster *tau* mutation within *CK1ε*.^89^ Mice homogygous for the *tau* allele (*CK1ε^tau/tau^*), like the hamster, display a shortened behavioral rhythm of ∼20 hours.^89^ The investigations would retain the presence of the clock––yet run clocks at different speeds!––in order to retain rhythmicity and also to promote internal desychrony through discordance of oscillator at varying speeds (e.g., 24 vs. 20 h). A phase difference of 8–12 h allows entraining of the SCN oscillator at different phases while driving other oscillators at opposite phases. These future studies should be able to dissociate the pleiotropic or intermingled mechanisms of circadian rhythms and their involvement in peripheral metabolism.

Overall, Loudon argued that the field has a biased view of circadian physiology and the involvement of the SCN as a master circadian pacemaker. Our research should expand to include other species and models besides nocturnal laboratory rodents, such as more diurnal species. The advent of better, more readily implemented genetics approaches, such as CRISPR, allows the field to broaden the exploration of circadian control of peripheral physiology.

### The compromise: peripheral oscillators are servants, but there is always more work to be done

Both parties agreed that peripheral metabolic rhythms are directly or indirectly regulated by the SCN, although this hierarchy needs to be more closely examined. A majority of our current understanding comes from laboratory rats and mice, and more amenable models (i.e., models from nature) may lead to novel discoveries and more complex understanding of the system. Shedding light on these issues also requires an update to how the field thinks about these questions and how we design future experiments to specifically interrogate the role of the SCN in peripheral metabolic rhythms and other physiology.

## Metabolism and mood: clinical studies

### Major depressive disorder and metabolic syndrome: the effects of antidepressant treatments

Major depression and metabolic syndrome are highly comorbid and independently constitute a significant public health burden. There is growing interest to understand the relationship between these comorbid disorders. Emmanuelle Corruble (Paris Sud University and Bicetre University Hospital) posed several questions regarding this association: (1) What is the impact of antidepressant treatment on metabolic syndrome in patients with major depressive disorder (MDD)? and (2) Are metabolic changes following the initiation of antidepressant treatment attributable, at least in part, to weight gain?

Corruble has made significant contributions to answering those questions with research from her own laboratory. Her laboratory recruited over 600 patients who were experiencing a major depressive episode from six psychiatric centers in France for a 6‐month prospective observational study. Patients were assessed at the initiation of antidepressant treatment and at 3 and 6 months later for depression symptoms and components of metabolic syndrome (defined according to the International Diabetics Federation definition).^90^ Antidepressant treatments were decided on a case‐by‐case basis by the psychiatrist. Patients differed on antidepressant monotherapy, which included selective serotonin reuptake inhibitors (39%), selective norepinephrine reuptake inhibitors (39%), tricyclic antidepressants (8%), and others (13%). Patients were excluded from the study if they displayed psychotic symptoms, had a diagnosis of bipolar disorder, misused drugs, had an eating disorder, or were pregnant or breast‐feeding. Patients were not excluded from participation for taking benzodiazepines or for participating in individual psychotherapy. There was an attrition rate of 62% from baseline to the 6‐month assessment. Those who dropped out of the study did not differ from completers on age, sex, lifetime duration of major depressive disorder, lifetime duration of prior antidepressant medication, Hamilton Depression Scale total score at baseline, current antidepressant use, presence of the metabolic syndrome at baseline, or tobacco use.

The study population consisted principally of women (69%) who were inpatients (87%) at the time of study. The majority (58%) were antidepressant free at the time of the initial evaluation. Only one‐fourth of the patients reported that this was their first episode of major depression. Those patients who met criteria for the metabolic syndrome tended to be older and to have a longer lifetime exposure to antidepressant treatment. Almost 30% of the sample met criteria for the metabolic syndrome at baseline. This number represents a high rate of metabolic syndrome compared with the 10–15% prevalence of metabolic syndrome found among the general French population. For the entire sample, depressive symptoms significantly decreased over the 6‐month observation period of the study. From baseline to 6 months, there were statistically and practically meaningful increases in components of the metabolic syndrome, including increases in waist circumference, systolic blood pressure, diastolic blood pressure, and fasting glucose, in the entire sample. There were also increases in high‐density lipoprotein cholesterol. Among patients with metabolic syndrome at baseline, there were significant increases at 6 months in systolic blood pressure, fasting glucose, and high‐density lipoprotein cholesterol. Among those patients without metabolic syndrome at baseline, there were significant increases in waist circumference, systolic blood pressure, diastolic blood pressure, high‐density lipoprotein cholesterol, and fasting glucose.

Notably, in the entire sample, the number of patients meeting criteria for the metabolic syndrome increased from the baseline assessment to 6 months. Importantly, these changes were independent of weight gain but were influenced by age at initial assessment. These results were able to answer the second question posed by Corruble at the start of her talk regarding the influence of weight gain over time on rates of the metabolic syndrome 6 months after initiation of antidepressant monotherapy. This large observational study had several notable strengths. This was a prospective study of patients currently experiencing a major depressive episode who were being treated with an antidepressant by their prescribing physician. The sample size was reasonable and allowed adequate time for important observations about changes in the components of the metabolic syndrome. The study was also able to account for a wide range of clinical and demographic variables. While other lifestyle factors, such as nutrition, physical activity, and sleep patterns, have yet to be examined, these results suggest that antidepressant treatment may induce or worsen metabolic syndrome in patients with major depressive disorder.

### Bipolar disorder and metabolic syndrome: the role of sleep

Chantal Henry (University of Paris) began her presentation on sleep disturbances and metabolic syndrome in patients with bipolar disorder by describing the differences between mood, emotional reactivity, and emotion regulation. Mood is a diffuse, fleeting state that lasts for hours, days, or longer. Emotional reactivity refers to the threshold and magnitude of a perceptual, behavioral, affective, or physiological response to an emotional stimulus. Emotion regulation is defined as the ability to respond to the demands of an experience with a range of emotions and in a manner that is socially permissible and sufficiently flexible.

Henry described an organizational dimensional framework for understanding subtypes of patients with bipolar disorder called the Multidimensional Assessment of Thymic States (MAThyS).^91^ Patients are classified according to five quantitative dimensions (i.e., emotional reactivity, cognition, motivation and psychomotor function, sensory perception, and interpersonal communication) and seven emotional valences (i.e., euphoria, happiness, irritability, panic, sadness, anger, and anxiety). One of the quantitative dimensions, emotional reactivity, is further broken down into normal emotional reactivity, emotional hyperreactivity, and emotional hyporeactivity categories. Patients belonging to the emotional hyporeactivity category are behaviorally inhibited and tend to have inadequately treated depression. Those patients categorized in the emotional hyperreactivity group tend to be mildly agitated and experience labile emotional states. When assessed for components of the metabolic syndrome and markers of systemic inflammation, patients in the emotional hyperreactivity group had statistically and practically meaningful elevations in systolic blood pressure, triglycerides, abdominal circumference, and C‐reactive protein, a marker of systemic inflammation.

Henry then provided a brief overview of the role of naturalistic and experimentally induced sleep disruption and emotional reactivity. Naturalistic sleep loss and experimentally induced sleep deprivation in the laboratory are key behavioral antecedents to labile affect, especially irritability. Neuroimaging studies of healthy participants show that sleep‐deprived participants showed larger amygdala responses and weaker functional connectivity between the amygdala and the prefrontal cortex while viewing emotional pictures.^92^ These findings among healthy participants have implications for understanding sleep disruption and emotional reactivity in remitted bipolar patients.

Henry described ongoing research in her laboratory aimed at understanding how subjective reports of sleep quality mapped on to the emotional reactivity subtypes of remitted bipolar disorder patients. When a larger cohort of patients with remitted bipolar disorder was categorized according to sleep quality on the Pittsburgh Sleep Quality Index (PSQI)^93^ and the emotional reactivity dimension of the MAThyS,^91^ three distinct clusters of patients were defined: those with poor sleep quality (PSQI ≥5) and emotional hyperreactivity, those with poor sleep quality and emotional hyporeactivity, and those with normal sleep quality and normal emotional reactivity. Those patients with remitted bipolar disorder who were classified as having poor sleep quality and emotional hyperreactivity had statistically and practically meaningful elevations in systolic blood pressure, abdominal circumference, and C‐reactive protein compared with the other two groups. Henry concluded her presentation by proposing a feedback loop among sleep deprivation, emotional reactivity, stress sensitivity, and metabolic disturbances as a framework for understanding metabolic changes that accompany bipolar disorder.

## Lessons learned

### Clinical science take‐home messages for basic scientists

Clinical science take‐home messages were presented by Michael Hastings (MRC Laboratory of Molecular Biology), who began by emphasizing the close affinity between circadian clocks and glucose metabolism and commenting that this relationship, which has likely developed from the evolutionary drive to make use of scarcely available resources, has been elegantly demonstrated throughout the meeting using both animal models and human data. He argued that, from a clinical perspective, the strongest take‐home message for basic scientists should be that people are still dying of metabolic disease––a treatable disease. Therefore, it is difficult to imagine a stronger motivation to explore the influence of the circadian clock on metabolism and physical illness.

Hastings commented that, although social and cultural changes over the past few decades have clearly contributed to the rise of metabolic syndrome, it is unclear exactly why we eat so much, an issue that may be better tackled by sociologists and/or psychologists, and which needs to be addressed at the governmental level through public health policy and interactions with large commercial enterprises. Biology research, however, is still vitally important in addressing these issues—for the time being, people will continue to eat unhealthily, and an understanding of the molecular mechanisms underpinning metabolic syndrome is therefore needed to aid drug discovery. It is also clear from both basic and clinical research into perturbed sleep, whether through sleep fragmentation, restriction, or acute misalignment, that profound rapid and reversible glucostatic consequences occur. What does this mean to a society in which individuals may have their sleep chronically restricted or misaligned, such as rotational shift workers?

Hastings also argued that basic researchers should keep the big picture in mind (i.e., although we must focus on individual proteins or neurotransmitters to uncover biological mechanisms and direct drug‐based approaches, this focus must not become so concentrated that attempts to address the wider problem are forgotten). Clocks are a systems‐level phenomenon and so can be used to understand and treat systems‐based diseases, such as metabolic syndrome. In turn, clinical scientists can aid basic research by making it clear exactly what the important questions to be addressed are.

There is a tremendous burden on clinical scientists who study and treat metabolic and psychiatric disorders to accurately define symptoms or categorical constructs related to disease and whether these contribute to disease risk (i.e., low versus high risk). Biologists also need to be clear about the clinical phenomena to model for investigation. Thus, guidance from clinical scientists is necessary for basic researchers to focus on the most pertinent or pervasive disease construct or cluster of symptoms. For example, sleep dysfunction may be common to both metabolic and psychiatric disorders or more relevant to a subcategory of patients. Identifying core symptoms, constructs, and/or endophenotypes of these diseases is necessary for stratification of patients in order to investigate the biology of disease and to maximize treatment options and outcomes.

Hastings suggested that sleep is an important clinically relevant indicator for understanding pathology associated with disease. He stressed that sleep characteristics are robust reporters of brain state, and, thus, any perturbation of brain state will be reflected by certain sleep phenotypes. However, sleep dysfunction may also be a contributor or causative factor to disease pathology. Sleep and circadian rhythms are biological, systems‐wide processes that likely have major roles in disease pathologies. Sleep is beneficial to the brain and to many physiological and cellular processes throughout the body.

Investigating the role of sleep and circadian processes in disease will be aided by the advent of new technologies in the clinic and the laboratory, especially developments in mobile phone applications and sensors to monitor and interact with patients in the real world. There is tremendous potential to continuously monitor sleep and other biological variables through these tools, which should provide a more comprehensive understanding of the role of sleep and whether sleep interventions are viable treatment options for certain diseases.

### Basic science take‐home messages for clinical scientists

Derk‐Jan Dijk (University of Surrey) followed by describing the expectations a clinical scientist from the basic research community has in studying the relationship between circadian rhythms and metabolism. The primary biological questions addressed during the meeting were (1) How is the molecular clock coupled to metabolism?, (2) What is the extent of circadian regulation of metabolic processes?, and (3) What are the implications for drug development? Dijk summarized the findings presented at the meeting from basic researchers and discussed the overall themes relevant for clinical scientists.

To address how circadian rhythms regulate metabolism, Peek's findings were highlighted, in which autonomous mitochondrial rhythms in oxidative metabolism were recorded and attributed to the molecular clock using a combination of fasting protocols and *Bmal1* knockout mice.^17^ By identifying the pathway by which this occurs, this research has implications for drug development, demonstrating the value of mechanistic biology to clinical researchers.

Dijk then discussed the research presented by Green, in which disruption of circadian control of mRNA polyadenylation influenced metabolism. Interestingly, genetic loss of the enzyme responsible for circadian polyadenylation resulted in resistance to diet‐induced diabetes and fatty liver. Beyond basic biology, these studies may remind clinicians to investigate genetic differences among individuals in order to identify mechanisms of resistance that could provide clues for treatment. For example, are there people who are resistant to the adverse consequences of shift work or other types of environmental circadian disruption?

The limitations of human circadian/sleep studies were then discussed. Specifically, it is common for study participants to undergo a single experimental challenge followed by assessments (e.g., sleep restriction). It may be more appropriate to expose an individual to multiple challenges, as experienced in the everyday world, which may help the dissection of the mechanisms underlying some of these adverse health consequences.

Returning to the molecular link between redox/metabolic oscillations and the molecular clock, Dijk referred to work presented by Reddy, who observed changes in the circadian period of mammalian cells and tissues and in fruit flies when core glucose metabolism is manipulated.^26^ This implies that metabolic pathways can feed back to and influence the core molecular clock mechanism. The question of which came first––the core molecular clock loop or a metabolic oscillator––is therefore a complex one. It may be, however, that the mechanism largely evolved as a complete system, and describing the system in its entirety may be more informative.

Investigation of this organism‐wide circadian system, Dijk remarks, has been attempted and presented in the meeting by Hogenesch, who looked at rhythmic gene expression across multiple organs. Although 43% of all protein‐coding genes showed circadian rhythms in transcription somewhere in the body, the composition of these transcripts varies between tissues.^94^ Temporal profiles of gene expression even differ within organs, with some having morning or evening “rush hours” of transcription (times of peak circadian gene expression) and others having both. The observation that 56 of the 100 best‐selling drugs target circadian genes have immediate implications for chronotherapy (i.e., we must note these temporal differences in order to target drugs to different organs in a circadian time‐dependent manner).

Dijk also highlighted an important new method of analysis described by Hogenesch, which can describe gene enrichment in rhythmic time series. It has previously been problematic to analyze periodic data, in which the beginning and end of a cycle are the same. Hogenesch has used this new method to analyze the impact of a restricted‐feeding diet in mice on liver gene expression, observing consistent and differential phase differences between different anabolic and catabolic processes. This work further demonstrates the complexity of the circadian system (i.e., desynchrony not just between organs but also within them).

Basic scientific researchers are steadily progressing toward a more comprehensive understanding of the bidirectional relationship between circadian rhythms and metabolism. Dijk suggested that clinicians should utilize these findings, in collaboration with basic researchers, to determine whether information could inform treatment or interventions. Previous research in humans has focused on desynchrony between the external and internal environments, but the data presented during this meeting suggest that this relationship is much more complex. If clinicians believe in this concept, there is a need to think hard about how monitoring the effects of various manipulations in human sleep or metabolic studies are interpreted.

### Audience comments on “lessons learned”

#### Marion Leboyer (University of Paris)

“It is clear that time matters, and we should therefore think about multiple‐hit models as well as epidemiological data that would help to implement several successive environmental factors so that we can gain a better understanding of what happens in real life. Exploration of the link between these environmental factors and genetic risk factors may lead to improved treatments.”

#### Andrew Loudon (University of Manchester)

“Pioneering the integration of mobile phone applications with drug systems in the clinic has great potential. One of the main problems in the development of chronobiology, however, is getting pharmaceutical companies to think outside the box. Furthermore, clinicians are not taught chronobiology and its implications in medical school, a comment supported by Ellen Frank, who noted that physicians are typically required to learn a great deal of information that may rarely have practical application in the treatment of patients, while the circadian system is likely to influence every disease they treat.”

## Summary

Over the past several decades, there have been dramatic changes in daily lifestyles, including factors that alter or disrupt sleep and circadian rhythms, such as an increase in shift work, ubiquitous artificial light, and enhanced work–life demands. These changes in the so‐called “social *zeitgebers*” have consequences for an individual's food consumption and metabolism. The relationships among circadian rhythms, metabolism, and disease are undoubtedly complex. The presentations of the 2015 INSPIRE meeting described the ways in which the circadian clock and glucose metabolism interact across multiple biological levels of the organism. These and other findings are providing the foundation for beginning to understand how circadian and/or sleep disruptions may lead to an increase in the vulnerability or predisposition to develop chronic diseases, such as cancer, and metabolic and psychiatric disorders. Understanding the basic mechanisms by which the circadian system regulates metabolism and how changes in energy state modulate the clock provides clues as to when and why these systems go awry. Sleep and circadian rhythms modulate, control, or regulate much of the mammalian physiology and thus are uniquely positioned for systems‐focused research. Sleep and circadian systems may be correlated with many diseases, but the challenge for future research is to determine whether sleep and circadian dysfunction are secondary to the disease, precede and are causal to the disease state, or are affected by the disease itself. From basic and clinical research, these are all plausible, especially for metabolic and psychiatric disorders. Indeed, circadian dysfunction may contribute to the onset of the disease, and the pathology may affect the circadian system, which may or may not contribute to the progression of the disease. Interventions, both environmental (e.g., social rhythm therapy or steady daily schedules) and pharmacological (e.g., compounds targeting specific components of the molecular clock), may have tremendous therapeutic potential. Moreover, timing of administration of these interventions should also be investigated and considered for treatment options. Chronotherapy (i.e., phase‐specific timing of pharmacological delivery) has been appreciated by oncologists and the cancer field for some time now. Similar treatment considerations may benefit other diseases by improving therapeutic efficacy and promoting recovery.

## Conflicts of interest

Dr. Frank serves as a consultant to Servier and Mitsubishi‐Tanabe and holds equity in HealthRhythms, Inc. and Psychiatic Assessments, Inc. No other authors have anything to declare.
